# Neuroprocessing Mechanisms of Music during Fetal and Neonatal Development: A Role in Neuroplasticity and Neurodevelopment

**DOI:** 10.1155/2019/3972918

**Published:** 2019-03-20

**Authors:** O. Chorna, M. Filippa, J. Sa De Almeida, L. Lordier, M. G. Monaci, P. Hüppi, D. Grandjean, A. Guzzetta

**Affiliations:** ^1^Department of Developmental Neuroscience, IRCCS Fondazione Stella Maris, Pisa, Italy; ^2^Division of Development and Growth, Department of Pediatrics, University Hospital of Geneva, Geneva, Switzerland; ^3^Swiss Center for Affective Sciences, University of Geneva, Geneva, Switzerland; ^4^Social Science Department, University of Valle d'Aosta, Aosta, Italy; ^5^Swiss Center for Affective Sciences and Department of Psychology and Educational Sciences, University of Geneva, Switzerland; ^6^Department of Clinical and Experimental Medicine, University of Pisa, Pisa, Italy

## Abstract

The primary aim of this viewpoint article is to examine recent literature on fetal and neonatal processing of music. In particular, we examine the behavioral, neurophysiological, and neuroimaging literature describing fetal and neonatal music perception and processing to the first days of term equivalent life. Secondly, in light of the recent systematic reviews published on this topic, we discuss the impact of music interventions on the potential neuroplasticity pathways through which the early exposure to music, live or recorded, may impact the fetal, preterm, and full-term infant brain. We conclude with recommendations for music stimuli selection and its role within the framework of early socioemotional development and environmental enrichment.

## 1. Introduction

The human brain is both wired with innate music abilities and shaped by music experience, starting in utero and continuing across the lifespan [1]. A growing body of literature from music therapy, music cognition, musicology, neurosciences, and affective and behavioral sciences target fetal and neonatal life, shedding light on the emergence and early development of sound and music perception. However, the great variability present in the literature in terms, for example, of type of music exposure, means of music administration, or age at exposure, has not yet allowed a clear understanding of how music experience impacts and shapes the human infant brain in the context of early neuroplasticity.

Human neural processing of music involves an extremely complex and widespread bilateral network of cortical and subcortical areas, integrating several auditory, cognitive, sensory motor, and emotional functions [2, 3]. Although part of the mechanism underlying music processing might be explained by simple sound processing, music perception is more than the sum of its basic acoustic features. In addition to auditory signal transduction, it triggers a sequence of cognitive, motor, and emotional processes that involve a number of brain areas, unilaterally (e.g., pitch and melody processing are more lateralized to the right hemisphere), as well as bilaterally, involving a number of “musical subfunctions” (for review see [4]).

The wide effects of music on brain function, encompassing auditory perception, language processing, attention and memory, emotion and mood, and motor skills, have suggested the use of music as a therapeutic tool in neuropsychiatric patients, including young infants at neurodevelopmental risk. Indeed, several systematic reviews and meta-analyses have examined the therapeutic role of music in preterm infants at neurodevelopmental risk, with inconclusive results, mainly due to the variation in study quality and methodology [5–10]. In most cases, the effects of the intervention were assessed in relation to cardiorespiratory parameters, growth and feeding outcomes, length of stay, effects on behavioral state, or pain. Much less is known on the effect of music intervention on direct measures of brain function and structure or on short- and long-term neurodevelopmental outcomes.

In this viewpoint review, we will firstly summarize current knowledge on the emergence and development of music processing during fetal and early postnatal life. Then, we will review the effects of music exposure in fetuses, preterm, and term newborns, with a specific emphasis on the effects of music on brain structure and function. Finally, in the light of these findings, we will discuss the possible role of music in early intervention programs, within the framework of early socioemotional development and environmental enrichment.

## 2. Emergence and Early Development of Music Processing in Fetuses, Preterms, and Term Newborns

### 2.1. Evidence of Music Processing through Behavioral Techniques

Studies measuring fetal movement or heart rate response to sound have shown that, although yet not fully mature, the developing auditory system enables responses to sound in utero from around 25 weeks of gestation. Fetuses respond first to low frequency 250 or 500 Hz tones, at around 25-27 weeks, and then to the 1000 or 3000 Hz tones by 29-31 weeks [11]. Fetal sound sensitivity, which refers to the intensity required to elicit a motor response (fetal movements quantified by ultrasound) at different frequencies, matures rapidly between 24 and 35 weeks of gestation [12]. High-intensity music to fetuses was shown to induce heart-rate accelerations and increased motor responses, whereas low-intensity music showed opposite effects [13]. An interesting study by Kisilevsky et al. [14] assessed the maturation of fetal response to music by evaluating fetal heart rate and fetal movement and suggested that a change in processing of complex sounds (such Brahms' Lullaby) might occur at around 33 weeks. At around term, by monitoring fetal cardiac responses, it was possible to show that near-term fetuses have very precise modulations of physiological behaviors related to specific aspects of the musical stimulus, such as sound intensity, frequency, and spectra [15–19] and can also process some fast and slow amplitude temporal variations in auditory streams [20].

A number of behavioral experiments appear to support the ability of newborns to process music per se. For example, preterm infants and term born neonates entrain to live-sung consonant lullabies, including synchronization of their sucking, mouth protrusions and tongue movement, respiration, and vocalizations which match the music contours [21]. There is compelling evidence that the ability of newborns to respond to music, and process it, is influenced by the sound exposure during the last trimester of gestation in the womb [22]. Newborn infants prenatally exposed to music and with minimal or no exposure to it after birth, already show, in the first days of life, physiological responses to music including reactions to basic rhythmic and pitch patterns. These fetal memories can affect different psychobiological domains, and their impact is carried into the newborn period [23, 24].

### 2.2. Evidence of Music Processing through Neurophysiological Techniques

Fetal auditory-evoked responses have been evaluated with magnetoencephalography (MEG) studies and identified from 27 weeks gestation, showing a continuous decrease in latency with age until term [25, 26]. In these studies, pure tones, single tones, or syllables have been the stimuli of choice while to date no MEG studies have used music. A recent study used seven different amplitude-modulated tones with carrier frequency of 500 Hz for the evaluation of the fetal (31-40 weeks) brain response to envelope slopes and intensity change at the onset of the sounds [27]. The authors found significant differences between the response latency to low, middle, and high rates of amplitude modulation, supporting fetal ability to differentiate between intensity changes of sounds and not only frequency changes.

In preterm infants, brainstem auditory-evoked responses appear at around 27 to 29 weeks of gestation, showing synchronous eighth-nerve activity and brainstem responses [28–31]. In keeping with fetal MEG studies, the absolute latencies decline progressively with advancing age and with an inverse relationship to the intensity of the auditory stimulus. In the following weeks, initial cortical-evoked potentials are noted and seen as a pattern of intermittence of low- and high-voltage activity. Low voltage is asynchronous and also referred to as “relative quiescence” while high voltage is already present simultaneously in the corresponding areas of both hemispheres and therefore, synchronous, beginning at the occipital lobe and moving forward to the temporal regions. Auditory-evoked potentials in premature neonates at 33 week gestation have shown early cortical activity with nearly mature biomechanical function of the cochlear signal. The lack of electrical activity in the olivocochlear system in premature neonates before 32 weeks gestation might indicate that before that stage the immature auditory pathways cannot relay the information from the periphery to the cortex [32, 33].

In healthy full-term infants, the innate ability to detect the beat in a music sound sequence has been investigated with EEG on day 2 or 3 of life. The stimulus used was a 2-measure rock drum accompaniment pattern composed of snare, bass, and hi-hat spanning 8 equally spaced (isochronous) positions. The authors concluded in favor of the existence of beat detection abilities, based on the expectation of pattern downbeat, as measured by peak amplitude measurements 200 ms before and 600 after the stimuli presentation [34].

### 2.3. Evidence of Music Processing through Neuroimaging Techniques

Few functional MRI (fMRI) studies have assessed fetal and neonatal response to sound. Studies evaluating fetal response to sound have demonstrated fetal hearing functional response to pure tones from 33 weeks of gestation in the left temporal lobe, localized between the sylvian sulcus and the superior temporal sulcus, consistent with the location of the primary auditory cortex [35]. Therefore, sound processing can already be observed beyond the reflexive subcortical level at the beginning of the third trimester. In full-term newborns, fMRI has revealed a bilateral BOLD (Blood Oxygen Level Dependent) response in the superior temporal regions to an auditory stimulation by a tonal sweep [36].

As far as music processing is concerned, two studies have evaluated near-term fetal response to music by fMRI. One used as stimulus is a Spanish guitar music, showing temporal activations in 4 of 7 participants and frontal activation in 1 participant [37]. The second one used as stimulus is a mother's voice singing a nursery rhyme, showing a significant temporal lobe activation in 2 of 3 fetuses [38].

In another study, one to 3-day old term born western neonates showed right lateralized auditory cortex activity as well as neural responses within the limbic system to altered musical stimuli when excerpts of western tonal music were used. In this study, western tonal music evoked predominantly right-hemispheric activation in primary and higher order auditory cortex. However, when altered versions of the same excerpts are presented, activation diminished in the right auditory cortex, instead emerging in the left superior and middle temporal regions, left inferior frontal cortex, and the limbic structures [39].

## 3. Effects of Music Exposure in Fetuses, Preterms, and Term Infants

Several studies have explored, in fetuses and newborns, the effects of the experimental manipulation of music stimuli to test the specific influence of music as compared to other or no stimuli, either between groups of otherwise matched subjects or within the same subjects at different times (intervention studies). The majority of them have focused on infants at neurodevelopmental risk, in particular preterm infants, as shown by the relevant number of systematic reviews and meta-analyses that addressed the question on the effects of music intervention in neonatal intensive care unit (NICU) populations [5–10]. In most cases, the effects of the intervention as to infant parameters were assessed in relation to physiological indexes such as heart rate or respiratory rate, to growth/feeding outcomes and length of stay, to impact on behavioral state, or to pain attenuation. In spite of the numerous studies available, systematic reviews of the literature failed to provide conclusive results on the benefit of music intervention in infants at neurodevelopmental risk, possibly due to the high study heterogeneity. Indeed, the reviewed studies have shown important differences in methodological aspects such as type and complexity of music exposure (e.g., vocal, instrumental, solo, or ensemble/orchestral), means of music administration (e.g., live or recorded music played in the environment; live or recorded music directed to/provided for each infant), and age or age range of the exposition. Much less attention has been given to the effects of music intervention on more direct measures of brain function and structure, which is the focus of this part of the present review.

### 3.1. Effects of Music Exposure as Assessed by Neurophysiological Techniques

Few studies investigated through neurophysiological techniques the effects of fetal exposure to music. In one study, fetal exposure to a simple recorded lullaby presented 5 times per week starting from the 29^th^ week of pregnancy until birth was compared to controls. The exposure group had significantly stronger amplitude event-related potential (ERP) responses at birth and 4 months that also correlated with the amount of prenatal exposure [40]. This indicates that prenatal music exposure has an effect on the neural responsiveness to sounds several months later, supporting a sustained effect of fetal memory through early infancy.

In preterm-born infants, amplitude-integrated EEG (aEEG, a restricted channel, compressed display EEG) has been utilized to investigate the effect of recording music on sleep-wake cycles, reporting positive effects of music exposure on quiet sleep in hospitalized premature infants [41, 42]. More recently, aEEG was used to study the effect of Brahms' Lullaby on the sleep-wake cycle of low-risk preterm infants between 33 and 37 weeks of gestation, reporting fewer interruptions of quiet sleep and increased postconceptional age sleep patterns as the result of music exposure [43]. The results, however, were called to be read with caution due to the potential conceptual flaws in the interpretation of the findings presented by the authors [44].

Additionally, ERP responses have been used as a biomarker of infant speech-sound differentiation during the neonatal period; at the same time, cortical responses to speech sounds were shown to be a feasible measure of the effect of infant vocal music exposure in the NICU. Specifically, infants were exposed to their mother's *a cappella* lullabies recording versus standard female *a cappella* lullabies recording, contingent to infant suck response for 20 minutes twice per day for 2 weeks. Infants in both groups had an increase in speech sound differentiation response on ERP. However, those that listened to their mother's voice had greater increase in spoken (standard) speech sound differentiation [45].

Clinical compatibility of care and research is an important factor to consider in data collection with vulnerable populations such as preterm infants; therefore, recommendations are consistent in encouraging the utilization of multichannel methods for a comprehensive view of the maturation process of preterm born infants, especially since the feasibility of acquiring electrophysiology data at bedside has been well established [46–48].

### 3.2. Effects of Music Exposure as Assessed by Neuroimaging Techniques

To the best of our knowledge, no studies have investigated the effects of music exposition through neuroimaging techniques during fetal life.

In preterm infants, cranial ultrasonography was utilized for the evaluation of the effect of music on development. A study using cranial ultrasonography evaluated the effect of a musical intervention during neonatal stay of extremely premature infants until they reached term [49]. Infants exposed to maternal sounds (speech, filtered reading, and singing voice, as well as heart beat), for about a month of cumulative daily 3 hours of stimuli, had a significantly larger auditory cortex bilaterally, but not in frontal horn neither in corpus callosum, as compared to control newborns receiving standard care in the NICU. However, the magnitude of the right and left auditory cortex thickness was significantly correlated with gestational age but not with the duration of sound exposure.

Only one study explored music processing in preterm infants at term-equivalent age using fMRI [50]. The authors showed that very preterm infants at term-equivalent age already distinguish between a known music and the same melody played on a different tempo. In this study, authors used psychophysiological analysis to show that, unlike preterm infants without previous music listening or full-term newborns, preterm infants who listened to music from 33 weeks of gestation until term equivalent age show an increased functional connectivity between the primary auditory cortex and the thalamus and the middle cingulate cortex and the striatum, when listening again to the known music. These brain regions have been linked to tempo, familiarity, pleasantness, and arousing music processing, suggesting that these abilities might be modulated by music exposure during the week preceding term equivalent age.

## 4. Music and Musicality in the Frame of Early Social and Emotional Development

Findings summarized in the previous sections of this review support the view that, starting from the very early phases of development, listening to music is far from a simple auditory experience, as it triggers a series of cognitive and emotional components with distinct and interconnected neural substrates [51]. Human brain imaging studies have shown that neural activity associated with music listening extends well beyond the auditory cortex involving a widespread bilateral network of frontal, temporal, parietal, and subcortical areas related to attention, motor functions, memory [52–54], and limbic and paralimbic regions related to emotional processing [55–57]. Music can therefore be a useful tool for infant multisensory stimulation [58–60].

Experiments on adult rodents proved that enriched environment, including auditory enrichment, stimulates cortical plasticity [61, 62]. In humans, imaging studies on adults suffering from traumatic brain injury, stroke, and degenerative diseases have shown that they benefit from the exposure to music with an enhanced memory functioning, attention focusing, motor regulation, and emotional adjustment [63–65]. Särkämö and colleagues [66] further showed that music listening after middle cerebral artery stroke induced a larger increase of grey matter volume in frontolimbic network, including orbitofrontal cortex.

Development of neural networks in the perinatal period is highly dependent on the intrinsic and extrinsic multisensory activity driving maturation of neuronal circuits. In particular, music during prenatal and early postnatal period in rats has been shown to modulate brain development in improving learning capacities [67–69]. As prematurity affects socioemotional development and its neural correlates, musical intervention, as framework for brain plasticity, has shown major impact on the reward system [70]. Music induces activity in limbic (e.g., amygdala and hippocampus) and paralimbic structures (e.g., orbitofrontal cortex, parahippocampal gyrus, and temporal poles), regions implicated in emotion generation and regulation and might therefore influence the maturation of socioemotional development [71]. Previous studies showed that full-term infants in the first days of life already show neural emotional responses to musical stimuli [39]. Recently, preterm infants were shown to benefit from enrichment of their environment in the form of audio recordings of maternal sounds with an increased cortical thickness in primary auditory cortex [49]. But, to what extent music during the early postnatal period in preterm infants can influence socioemotional development and the underlying corticolimbic network formation?

Early social interactions in a specifically structured context, such as music and singing, can be a tool for early social and emotional intervention in a broad sense. Beyond the enrichment of auditory skills, through the organization of primary and secondary auditory brain regions, early experiences in music and singing might be also a way to sensitize newborns to the dynamics of social interactions. Actually, when parents interact with their newborns, they provide a dynamic structure in which microevents produced by parents (e.g., silence and prosodic accents) are contingent to the specific reactions of their newborns. For example, it has been shown that when preterm newborns produced a motor action such as open eyes or mouth corners elevation (interpreted as a smile by the mothers), the mothers modulated their voice in a dynamic way, thereby establishing a dyad contingency [72]. In this early face-to-face interaction, when infants open the eyes or smile, the maternal voice is perceived by adult naïve listeners as more emotional and more smiling than it is in the absence of any infants' facial display. When mothers sing or speak directed to their preterm infants, their vocal act is not only related to preterm infant behavior but also bears modulated emotional content [73]. It is likely, given the fact that music and singing induced brain activations in a widespread neural network including the reward and habits system, i.e., the basal ganglia, the orbitofrontal regions, and deeper structures including the amygdala and the hippocampus [74], that early interactions have an impact on the development and the organization of these neural networks involved in social interactions and emotion regulation. Moreover, it has also been shown that human voice induces a specific set of brain activations localized in the middle superior temporal sulci and gyri in adults, i.e., the so-called voice sensitive areas [75]. These regions have also been shown being sensitive to the emotion conveyed by the voice [76, 77].

Therefore, early exposure to human voice (often emotional in the context of early interactions between preterm babies and parents) in a structured context such as in singing might be served in neural organization during early stages of development and might have a long-term impact on the relevance and importance of human voice in social interactions. These voice-sensitive regions (i.e., superior temporal sulcus and superior temporal gyrus) are in close interactions with frontal areas and especially important for the acoustical invariance extractions. Therefore, premature infant exposure to voice during live interactions in early period may be a way to establish the precursors of the acoustical invariance extractions for the categorization of specific emotions inferred from human voice [78]. These abilities are fundamental in social interactions and especially important in the social exchanges between parents and fragile infants.

## 5. Where to from Here? Possible Role of Music in Early Intervention Programs

From the standpoint of the neurodevelopmental role of music in the NICU, the primary aspect for this mechanism is the environmental enrichment of sensory experiences of the preterm-born infant. Even in the absence of overt neural injuries and its severe developmental consequences, most infants born prematurely experience neurodevelopmental differences, many of which are believed to be a consequence of their stressful, atypical early sensory extrauterine hospitalization experience [79–81]. Many NICU environmental guidelines have been published and implemented in units caring for preterms; however, the simulation of the intrauterine environment is not feasible under current medical conditions. Therefore, the goal of environmental improvement is the inclusion of positive experiences to aide parent-infant bonding, infant sleep, and enriched awake hour interactions in the context of the medical reality.

Some of the most commonly reported music types that have been used in NICU studies are lullabies [59, 60, 82, 83]. Lullabies can have different structures and forms according to the different cultures they belong to, but they share several similarities that result from the intended audience and function of the songs [84]. The most frequently used lullabies present slow tempo and frequent repetitions, with few dynamic changes, with a limited pitch range and a high degree of continuity. These essential characteristics are shared by the other music pieces, such as Brahms or Mozart lullabies, which are also frequently used in studies. In addition, the reported music presented to preterm babies includes consistent consonance.

Unfortunately, except from studies with live music, the recorded music used in research with infants is rarely described in detail. While the intensity and duration descriptions are often accurate and report, respectively, the range levels in decibels and the precise duration in seconds, other musical components lack accurate descriptions. Further, the same music stimulus (i.e., acomposition extract) can be performed in different tempos, instrumentation, and arrangement, but this is rarely described.

In general, independent from the musical piece, the recorded music chosen for preterm infants show a high degree of similarity in the structure such as slow tempo, high levels of consonance, high number of repetitive elements, few sudden dynamic, pitch or timbre changes, and a limited pitch range [85, 86]. The main difference between the chosen extracts can be the presence or absence of vocals, even if previous studies did not find specific differential effects [85]. Further research is warranted to determine if the vocal component of the presented music, which makes the piece more comparable to the natural condition of maternal singing, can constitute a valuable variable in the efficacy of music exposure.

### 5.1. Recommendations for the Application of Music for NICU Neonate Population

The role of all developmental therapists, including those directly providing, or training parents to provide music to infants in the NICU, is first and foremost to provide parent support. This includes reading the infant cues and modeling appropriate interactions with the infant, especially in cases of severe medical fragility, high parental stress, and grief during the NICU stay. Therefore, recommendations are for live experiences actively involving parents as the most beneficial form of intervention for the overall neurodevelopment of infants and family support. In this respect, it is important to consider the variability in the hospital policies for parental visits to the NICUs, as some still do not allow 24/7 access to the infants even for parents. Visits are also based on maternity and paternity leave allowances and capabilities of parents to visit from remote locations. Therefore, recorded maternal/paternal voices and therapist provided experiences can be effective options in such circumstances.

Training of therapists providing or supporting music experiences for patients and families in the NICU is highly variable based on the country program structures and requirements [87, 88]. However, it is recommended that developmental specialists providing music experiences in the NICU or consulting music research with infants should be trained, at minimum, in infant development, music, physiology, medical terminology, and psychology.

At term and postterm, medically and neurologically stable infants (including infants maintained in the hospital) are able to tolerate and may benefit from continually more complex musical stimuli with increasing age, including vocal and instrumental (also orchestral) music. However, the exposure should be intentional. Since neonates have little awake time, the usage of this time to actively engage is recommended. Layering recorded music with movement/dance, tactile/massage, and visually stimulating (nonscreen) multisensory input is appropriate and recommended in later stages of development, when the repertoire of activities and infant's “readiness” for more complex sensory processing are expanded. Specifically, it is the opinion of the authors based on the previously published evidence, that music intervention provided in the NICU should be consistent with the following recommendations.

The effects of a music interventions—both live or recorded, administrated by parents or trained specialists—on very preterm infants with severe health complications or with very immature brains are currently unknown. Most of the studies in the previously reported systematic reviews and meta-analyses evaluating the effects of music interventions are limited to stable preterm infants. Thus, we recommend considering the stability and the age as key criteria for beginning a music intervention. Moreover, music experience for preterm and full-term infants has to be accurately planned with a trained developmental specialist. Similar to all of the early interventions delivered in the NICU, the music administration should consider infant needs and behavioral state. There is no evidence demonstrating that a specific music (i.e., Mozart Sonata) is beneficial per se for every infant and without an individualized and intentional professional intervention [89].

Every music intervention should respect the sound level guidelines for NICUs. The guideline was published by the American Academy of Pediatrics recommending for the combination of continuous background sound and operational sound to not exceed an hourly equivalent continuous sound level of 45 decibels [90].

The types of music that have been tested and most frequently reported in the previously mentioned systematic reviews include a limited pitch and dynamic range, an absence of sudden change (increase or decrease) in intensity, a slow tempo, and frequent repetitions.

Live music, directed to an individual infant [91], either administered by singing parents or supported by professionals, is primarily suggested as an early music intervention in the frame of environmental enrichment and infant emotional development [91, 92]. A recorded music administration should follow the principles of increasing in structure complexity and individualization to infant's reactions. With the increase of age, following a careful individual observation, the administered music can present further degrees of acoustical complexity, such as multiple instrumentation, a moderate degree of variation or length.

## 6. Conclusions

The neuroprocessing mechanisms of music stimuli during fetal and neonatal developmental stages warrant much more vigorous research with neuroimaging, neurophysiology, and behavioral techniques. The combined inclusion of these methods in robust randomized controlled trials is needed to evaluate both (i) the neuroprocessing mechanisms of music stimuli and its similarities or differences with auditory processing of other stimuli, such as language or voice, and (ii) the effects of music experiences and interventions on the developmental trajectories of the most vulnerable neonatal population: preterm-born infants cared for in NICUs. Robust clinical trials with specific attention to the differences between a live or a recorded music administration are needed. Similarly, studies are needed to investigate the specific effects of different types of administered music, instrumental versus vocal music and maternal singing versus other voices or music. Current evidence is, however, already available to support the utilization of music interventions in the context of environmental enrichment and family centered care for hospitalized infants.
